# Towards transformative WASH: an integrated case study exploring environmental, sociocultural, economic and institutional risk factors contributing to infant enteric infections in rural tribal India

**DOI:** 10.1186/s12889-021-11353-z

**Published:** 2021-07-06

**Authors:** Julia Vila-Guilera, Priti Parikh, Hemant Chaturvedi, Lena Ciric, Monica Lakhanpaul

**Affiliations:** 1 Population, Policy and Practice, UCL Great Ormond Street Institute of Child Health, London, WC1N 1EH UK; 2 Engineering for International Development Centre, The Bartlett, UCL Faculty of the Built Environment, London, WC1H 0QB UK; 3 Aceso Global Health Consultants Ltd., Chanakya Place 1, New Delhi, 110059 India; 4 Healthy Infrastructure Research Group, UCL Department of Civil, Environmental and Geomatic Engineering, London, WC1E 6BT UK; 5 Whittington Health NHS Trust, London, N19 5NF UK

**Keywords:** Enteric infections - Faecal exposure - infants and young children- sanitation and hygiene, Socio-ecological determinants, Rural India

## Abstract

**Background:**

Despite clear linkages between poor Water, Sanitation, Hygiene (WASH) and enteric disease, the design of effective WASH interventions that reduce child enteric infections and stunting rates has proved challenging. WASH factors as currently defined do not capture the overall exposure factors to faecal pathogens through the numerous infection transmission pathways. Understanding the multiple and multifaceted factors contributing to enteric infections and their interconnectedness is key to inform future interventions. This study aimed to perform an in-depth holistic exploration of the environmental, socio-cultural, economic and institutional context surrounding infants to develop an integrated understanding of enteric infection drivers in rural tribal Banswara, in Rajasthan State, India.

**Methods:**

This study relied on the triangulation of mixed-methods to capture critical influences contributing to infant enteric infection transmission. We conducted structured observations and exploratory qualitative research across 9 rural tribal villages, including transect walks, household observations, interviews with frontline health workers and group discussions with mothers. The emergent social themes and identified factors were mapped based on the scale of agency (individual, family or community-level factor) and on their nature (environmental, socio-cultural, economic and institutional factors).

**Results:**

Infants aged 5 to 24 months were seen to have constant exposures to dirt via mouthing of soil, soiled hands, soiled objects and food. Rudimentary household environments with dirt floors and domestic animals lacked a hygiene-enabling environment that hindered hygienic behaviour adoption. Several unsafe behaviours failing to interrupt infants’ exposures to pathogens were captured, but caregivers reported a lack of self-efficacy skills to separate children from faecal exposures due to the rural farming environments where they lived. Conceptual mapping helped understand how wider-level societal factors such as socio-economic limitations, caste inequalities, and political corruption may have trickle-down effects on the caregivers’ motivation and perceived self-efficacy for improving hygiene levels around children, highlighting the influence of interconnected broader factors.

**Conclusions:**

Conceptual mapping proved useful to develop an integrated understanding of the interlinked factors across socio-ecological levels and domains, highlighting the role of wider sociocultural, economic and institutional factors contributing to infant’s enteric infection risks. Future WASH interventions are likely to require similar integrated approaches that account for the complex factors at all levels.

## Introduction

Enteric infections are still responsible for 1.7 billion cases of child diarrhoea every year [1]. In addition, continuous diarrhoeal events and asymptomatic enteric infections during the first 1000 days of life, a critical developmental stage, are associated with long-term morbidities such as undernutrition and child stunting [2]. India, and particularly its rural areas with poor access to water, sanitation and hygiene (WASH), account for almost one-third of all the child stunting cases and one-fifth of child diarrhoeal deaths worldwide [3, 4]. Significant work remains to achieve the 6th UN Sustainable Development Goal (SDG) of basic WASH access for all by 2030 and the Government of India’s target to reduce child stunting to 25% by 2022 [5]. However, despite clear linkages between poor access to WASH and enteric disease, the design of WASH interventions that block infection transmission pathways and reduce child diarrhoeal and stunting rates has proved challenging [6, 7].

Traditionally, WASH interventions were designed in a top-down supply-driven approach that largely focused on latrines and clean water infrastructure provision [8, 9]. Since the turn of the century, the WASH development sector has seen a paradigm shift that encouraged the development of alternative approaches with an emphasis on community participatory action research [10] and integrative thinking across sectors [11–13]. Research has also started to recognise the need to address infant-specific risk factors, given that the first 2 years of life are a critical window for intervention [14]. Infant-specific infection risks that had been underexplored include factors such as the unsafe management of child faeces [15, 16], unsafe weaning food hygiene practices [17], soil ingestion and mouthing of soiled hands due to a lack of a safe play space for infants [18–20], and the inadequate separation of domestic animals that contribute to the infant’s exposure to animal faecal pathogens [21–23]. The development of a more comprehensive understanding of the enteric pathogen exposures for infants informed the design of recent WASH trials targeting infant-specific exposures with renewed optimistic prospects. These recent trials integrated the supply of WASH infrastructure and behaviour change strategies with the aim of addressing the multidimensional factors contributing to infection risks for infants. However, results from these high-profile trials [24–26] mostly failed to significantly reduce child diarrhoeal and stunting rates. These results spurred debates amongst researchers in the field, which concluded that future work needs to include *transformative* WASH interventions that are comprehensive and tailored to the local exposure landscape [27]. While it is still unclear what *transformative* WASH will need to entail, it has been argued that it will need to be a comprehensive package of WASH interventions that are context-specific, risk-based, and developed using participatory and Human-Centred Design methods [28, 29].

One hypothesis for these unsuccessful findings is that these recent trials were a step in the right direction but still insufficient to address enough key infection risks to influence child health outcomes due to the high faecal contamination levels and the existence of numerous infection transmission pathways in these settings [30]. Despite efforts in recent trials to tackle material needs as well as caregivers’ hygiene behaviours [31], the complexity of poor WASH settings, often linked to poverty in resource-limited areas leads to a myriad of environmental, socio-cultural, economic and institutional factors that impact and contribute to infants’ enteric pathogen exposures and stunting [32]. In this scenario, a gap remains to better understand the wide range of factors contributing to infections (environmental, socio-cultural, economic and institutional factors) and how they interact to shape the immediate pathogen exposures to infants. We hypothesised that a holistic exploration of the infants’ surroundings to develop an integrated understanding of the multiple components contributing to enteric infection in infants might shed some light on what future *transformative* WASH programming may need.

Given the complexity of WASH, with influences across different scales (i.e. individual, societal, governance …) and domains (i.e., infrastructure, behaviours, norms..), several frameworks such as RANAS [33] or IBM-WASH [34] have been proposed to organise the multiple factors that affect WASH behaviours. The socio-ecological model is another framework that has long been used to understand the complex and interrelated factors determining health outcomes at the multiple scales of agency: the individual, household, community and societal levels [35]. The socio-ecological model allows researchers to map the structural factors at the community and context-level operating through intermediary factors at the household-level to impact individual health [36]. A systems approach emphasises the need to assess the interconnectedness of the elements of a system to understand systems as a whole [37].

India’s north-western states such as Rajasthan have the highest under-5 diarrhoea mortality rates [38]. Within Rajasthan, the district of Banswara is one of the few remaining tribal districts, where over 50% of children are stunted [39]. This study was set to explore the infants’ surroundings in-depth to identify the wider range of factors contributing to enteric infections in infants (0–2 years), using rural tribal villages in Banswara as a case-study site. Following a systems approach and grounded in the socio-ecological model, environmental, socio-cultural, economic and institutional factors at the individual, household and community-level were investigated. Mixed-methods of data collection were employed and conceptual mapping was used to provide an integrated visual representation of the findings. Ultimately, we aimed to identify potentially overlooked enteric infection factors to guide future WASH programming design and advance research towards *transformative* WASH.

## Methodology

### Study design

A systems approach was adopted to enable an in-depth exploration of the myriad of enteric infection drivers across the socio-ecological levels. The elements of the system were defined as the multiple factors contributing to infant enteric infection. A case-study design focusing on small communities of less than 1500 people allowed us to better define the systems’ boundaries. Bringing together an interdisciplinary team comprised of public health, social sciences, microbiology, and civil and environmental engineering experts, allowed us to identify enteric infection factors from diverse natures. The PANChSHEEEL project [5] explored the linkages between health, education, engineering and the environment to identify the multiple determinants of suboptimal infant feeding at the household, community and governance levels in rural Banswara and co-develop an integrated intervention package. The proposed package components included a bundle of workshops and village activities such as knowledge building and demonstration & practice meetings, support groups, video shows, wall paintings and posters, as well as cooking classes and recipe books to improve infant and young child feeding and care practices [40]. Infant feeding practices and enteric infections are the two major contributors to the child undernutrition and stunting burden [41]. To get a more holistic picture, we built on the identified determinants of infant feeding under PANChSHEEEL [5], and further investigated the wider determinants of infant enteric infections across the socio-ecological levels, using the same integrated, interdisciplinary approach.

### Study setting

This research was conducted in the study communities within the PANChSHEEEL project. The study was carried out across 9 rural tribal villages in two community blocks of Banswara district, from September to December 2019. These two blocks were selected to represent district diversity. The Ghatol block is a canal irrigated, close to the urban capital area, and the Kushalgarh block is in a semi-arid, non-canal irrigated remote area. Banswara is a rural tribal district where 93% of its population is rural, and 77% pertains to Scheduled Tribes and Scheduled Castes (ST/SC) [39], the lowest socio-economic castes with higher poverty rates. Rural villages in Banswara are comprised of agricultural land and groups of household compounds organised in dwellings. Dwellings often gather around a common yard with a shared public water source. One to three public primary schools and one to two rural childcare centres are available in each village. The latter provide a hub for frontline health workers to deliver basic maternal and child healthcare services. Village municipalities (Gram Panchayats) are run by Sarpanchs (village political leaders), who receive funds from central government-sponsored schemes and state government ministries to provide basic amenities and aids for rural development programs.

### Data collection methods

Mixed methods were used in this research to allow for the triangulation of findings from different methods. Quantitative methods included a household survey, structured observations of the infant’s mouthing contacts at home, and structured observations of specific caregiver domestic practices such as cooking, cleaning, and handwashing. Qualitative data was collected using different participatory learning and action tools [42] including Transect Walks, Focus Group Discussions (FGD), Key Informant Interviews (KII), and unstructured household interviews and observations. Pictures were also taken to initiate topical conversations.

#### Transect walks

Transect walks are a participatory appraisal method that consist of systematic walks through a defined community area alongside the local people, to explore WASH conditions [10]. Transect walks enabled us to understand the village-level water and sanitation resources, how the community used and perceived them, and how they could contribute to enteric infection transmission. Nine transect walks were conducted, one in each of the study villages, and lasted from 2 to 5 h each. Guided by the community champions, village water sources, drains, schools, child-care centres and public toilets were visited, while informal conversations were held with the local school-teachers, frontline health workers and other community members. A semi-structured tool was employed to capture the physical context, and detailed field memos and pictures were taken, capturing the social interactions observed, and personal experiences perceived.

#### Key informant interviews and focus group discussions

The purpose of KII and FGDs was to understand community’s concerns regarding infant enteric infection risks, as well as capturing the local socio-cultural factors and norms for hygiene and childcaring habits. Semi-structured topic guides were developed to follow a consistent structure but to allow participants to elaborate in-depth on the topics discussed. Topic guides were developed following preliminary discussions with community champions during a previous piloting trip carried out in March 2019, where the main topics of particular concern for the community in regards to child health and infections were identified. FGD and KII were facilitated by local fieldwork researchers in the local language, a mix of Hindi and *Wagdi*, and observational notes and field memos were also taken (JVG). Audio recordings were transcribed verbatim and translated to English.

#### Household visits

Household visits allowed us to collect quantitative structured data on the infants’ mouthing contacts, the caregivers’ domestic hygiene practices, and the household’s built environment in the household survey, as well as qualitative data from unstructured household interviews and observations. A total of 63 structured observation hours (average 90 min per visit) during morning times (8 AM to 12 PM, due to feasibility reasons), were carried out. Household visits began with informal introductions, explaining the purpose of the study and obtaining consent. This was followed by a guided house tour, where participants walked us through their household compound, and the household survey was completed. Structured observations were then conducted capturing the infant’s mouthing behaviours and the domestic hygiene practices performed by the caregivers. To reduce reactivity bias, the local fieldworkers that had good rapport with the community members carried out informal conversations with the household heads while another researcher (JVG) annotated observational data. The household survey structured tool was based on the WHO/UNICEF WASH core questions tool [43]). Children’s mouthing behaviour-tallies and caregivers’ domestic hygiene practices were logged into a semi-structured observation tool (amended from the SHINE trial tools [18, 20], that were kindly shared).

### Participant recruitment

The fieldwork team consisted of the lead author plus two fieldwork researchers that were familiar with the local setting and languages, and already had regular contact and good rapport with the rural communities. Having conducted research in the same setting previously (during PANChSHEEEL), 2–3 focal individuals in each village (community champions) had been identified to help recruit participants and liaise for other field arrangements. In each study village, there were 1-to-2 frontline health workers with a designated role in WASH promotion and child infections and they were all purposively recruited for Key Informant Interviews (KII). A total of 27 mothers of young children were purposively recruited by community champions to conduct Focus Group Discussions (FGD) with 6–9 participants each. Lastly, 42 households with at least one under-2-year-old infant were purposively selected from each different dwelling within a village, to capture households with geographical diversity. Overall, 12 KII, 4 FGD, 9 transect walks and 42 household visits were conducted. Purposive sampling can lead to a biased sample but given the exploratory nature of the study and the observed homogeneity between village dwellings with similar material circumstances, socio-economic, and lifestyle factors, it was not expected to impact the general conclusions of the study.

### Data analysis

Quantitative data collected through structured observations and household surveys was entered into a Microsoft Excel sheet. It was then checked for completeness and analysed using descriptive statistics. Qualitative textual data from the KII and FGD verbatim transcripts, and from the transect walk, and unstructured household interview field memos were included for analysis using the NVivo software. Thematic analysis was used to identify analytical categories that were derived inductively from data. Analytical categories (themes) described physical or social phenomena providing an explanation as to how and why an enteric infection risk may occur in this community. Themes were classified based on the scales of agency (individual, family or community-level factor) and based on the discipline nature of the factor (environmental, socio-cultural, economic and institutional factors). Two researchers (JVG and HC) independently labelled potentially relevant statements which were grouped into preliminary themes. Then, researchers revised the codes together and disagreements were discussed to decide on the final set of overall themes that explained the main drivers of infant enteric infections identified.

#### Conceptual mapping

Based on the themes identified across the different levels of agency and domains as drivers to infant enteric infections, a conceptual map was drawn to visually represent the themes in an integrated manner. Conceptual mapping is a useful tool to visually represent the web of interconnected and complex factors and may aid in understanding emergent patterns when looking at the system as a whole [44]. The conceptual map was drawn in a sequential manner: at the bottom of the map, the most immediate exposure pathways to infants are represented (the individual-level factors), which are then sequentially linked to factors that were identified at the household, community and societal levels. In this manner, as the reader moves up the conceptual map, a collection of interlinked concepts aim to answer how and why infants may end up being exposed to faecal pathogens.

Triangulation happened at the interpretation stage so that findings arising from different methods could be compared for agreements and discrepancies [45]. To triangulate findings, results from different methods are presented and interpreted together, so that social themes from the analysis of qualitative data are substantiated by the descriptive analyses of quantitative data and visual images.

### Ethics

Study details were explained in the local language by local fieldwork researchers and written informed consent was obtained from all participants or, in the case of under-18 s, their legal guardians, before data collection started. No person identifiable data was taken except for pictures, for which separate consent was obtained and they were securely stored. The study was approved by UCL Research Ethics Committee in the UK (14,703/001), as well as the Institute of Health Management Research Ethics Committee in India (04052019/01).

## Results

Addressing the study aim of exploring infant enteric infection drivers in-depth, the themes presented provide thorough explanations of the environmental, socio-cultural, economic and institutional factors identified contributing to enteric infections. Themes were substantiated with integrated field data (interview quotes, pictures, observational data …). A conceptual map is then presented, distilling the detailed thematic descriptions and interlinking the concepts providing explanations as to why and how infants end up being exposed to faecal pathogens, allowing a holistic understanding of the system.

### Individual-level factors: Infant’s immediate faecal exposure contacts

As infants in their first 2 years of life progressed through the different developmental stages, they became increasingly exposed to potential faecal pathogens through dirt ingestion in their household environments, as well as through contaminated water and food pathways (Table 1). In the 42 households observed with at least one under-2-year-old, a total of 47 children were followed to capture their mouthing tallies: 11 of them were newborns (~ 0–5 months) without mobility, 13 could crawl and sit but not walk (~ 5–12 months), and 23 were fully mobile and able to walk (~ 12–24 months). Newborns that were still immobile were observed to be exposed to only two different locations: the caregivers’ arms and their beds. Semi-mobile infants were often placed on the household dirt yards to play and crawl (observed in 8 of the 13 infants in this category). Fully mobile infants’ play areas further extended around the house dwellings and they were sometimes seen playing in the animal spaces (observed in 6 instances) or drains (seen twice). The level and types of exposure to faecal pathogens changed significantly through their developmental phases. Mouthing of own fingers and having flies on the face were the exposure contacts that remained most prevalent across all infants at all ages (Table 1).

**Table 1:** Percentage of infants observed to be exposed to different pathways during household observations, by categories of mobility levels

Elements ingested or in contact with the child’s mouth	Immobile infants (*N* = 11)	Semi mobile infants (*N* = 13)	Mobile infants (*N* = 23)
Flies observed on lips and face mucus	64%	69%	74%
Mouthing of own fingers	100%	100%	100%
Mouthing of own fingers immediately after hand contact with dirt	0%	77%	74%
Mouthing of mother and sometimes grandmother’s breasts, during breastfeeding or as a pacifier method	73%	54%	39%
Mouthing of cloths, which were typically women’s *sari* scarfs and often visibly dirty, used as bed linen to wrap young infants	45%	8%	0%
Mouthing of miscellaneous objects in the environment, mainly visibly dirty plastic or plant elements	0%	62%	61%
Ingestion of food items while sitting on the floor with constant floor-hand-mouth contacts	0%	31%	57%
Direct ingestion of soil	0%	15%	39%
Ingestion of drinking water	9%	23%	35%

### Household-level environmental factors: households’ material circumstances

Material circumstances in households were observed to be generally poor, contributing to poorer food hygiene, personal hygiene, and general domestic hygiene conditions influencing the immediate space where children were exposed to the majority of their time during their first 2 years of life.

**Fig. 1:**
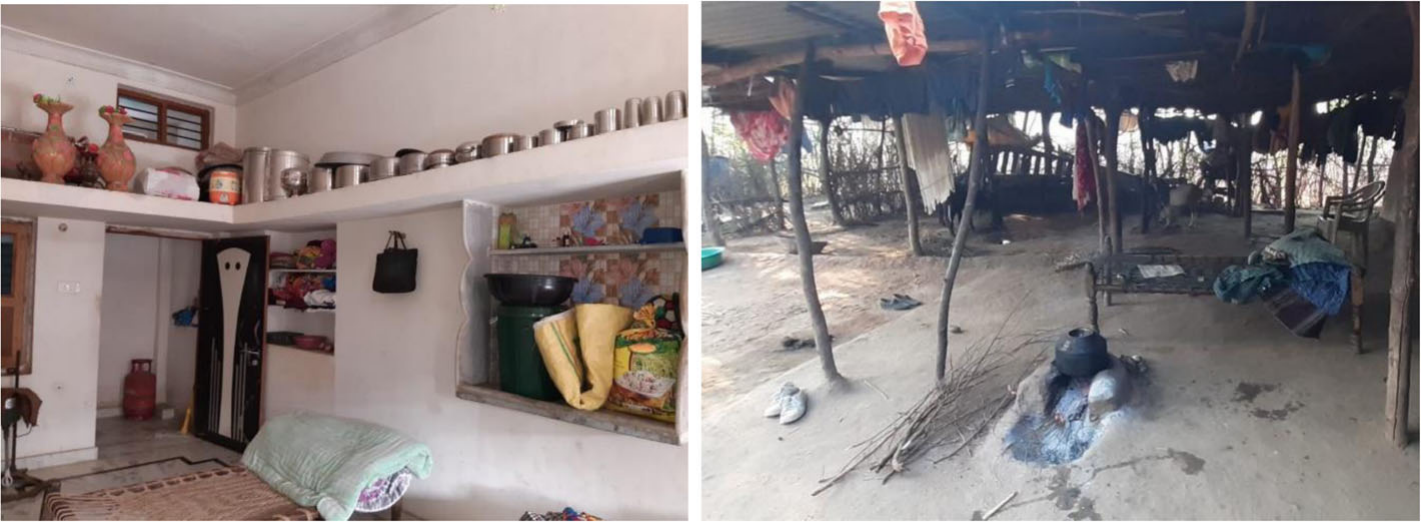
Living room in a Pucca household (left) and a Katcha household (right)

Typical house buildings in study villages were semi-permanent structures, made from rudimentary materials, with floors and walls typically plastered with a mix of mud and cow dung (Table 2). Houses of these characteristics were locally called *Katcha* houses, in contrast to the *Pucca* houses, which were made from higher quality finished materials such as cement and tiles (Fig. 1). The availability of tiled floors (instead of dirt floors) in *Pucca* households allowed for floors to be swept and mopped, improving the hygiene of a surface that infants had regular contact with while crawling.

**Table 2:** Material circumstance of the study households

Household material circumstances	Overall (*n* = 42)
*Katcha* houses	36 (86%)
Gas-stove ownership	18 (43%)
*Main water source*	
Public hand-pump	32 (76%)
Private electrical borewell	8 (19%)
Open unimproved well	2 (5%)
Distance to main water source > 100 m	16 (38%)
Storage of drinking water in the premises	41 (98%)
Storage of hygiene water in the premises	16 (38%)
Some type of latrine construction present	17 (40%)
Improved latrine available	5 (12%)
Livestock ownership (goats, cows, buffaloes, and bulls)	41 (98%)
Poultry ownership (chickens)	13 (31%)

While several houses had gas-stoves for cooking, families mostly relied on clay-stoves placed on the floors. Biomass from surrounding land and dried cow dung cakes were used as fuel for the clay-stoves.

The majority of the study households owned livestock animals, mainly cows and goats (Table 2), which were kept tied in the house courtyards. Only a few families, those that were not strict vegetarians by religion, were observed to have chickens, which roamed freely around the compound. *Pucca* households had designated separate animal sheds, but families in *Katcha* households kept animals around the children’s play spaces and often slept in the same room.

As a water source, most households used groundwater supplied by public hand-pumps, but a few families temporarily used unimproved open wells when groundwater was scarce. Drinking water was collected every 1–2 days from the source and stored in covered containers in the houses. Where the water source was relatively far from the house (> 100 m), families also collected water for other domestic purposes such as washing hands or kitchen utensils, which was stored in separate uncovered containers kept in the house yards. *Pucca* households instead mostly had a private water source at home, typically from electrical borewells, which allowed for easier access to water for drinking and domestic purposes.

Several houses had some type of latrine construction, but very few (and mainly *Pucca* houses only) had a safely managed improved latrine, as described by the WHO/UNICEF [46] (Table 2). Most of the latrine constructions observed were only partly completed, meaning that the constructions included latrine sheds (walls and roof) and latrine slabs, but the underground latrine pits for excreta disposal had not been constructed, thus, families still mostly relied on open defecation and the disposal of infants’ faeces in the near open environment.

### Household-level socio-economic factors: caste stratification of household material circumstances

Household material circumstances were reported to be largely dictated by the economic means of the families, which were distinctly stratified by social castes. Marked differences between tribal caste families and higher caste families within the study villages were observed. All higher caste families were seen to have higher socio-economic circumstances that led to improved household infrastructures including *Pucca* households with cemented floors, private water borewells, separate animal sheds, electricity and other features that influenced domestic hygiene levels. On the other hand, tribal caste families reported facing financial limitations to improve their WASH and material circumstances, such as not having enough space or money to build designated animal sheds, nor improved latrines or private borewells. Despite toilets being perceived as a modern feature, distinctive of higher socioeconomic status, major reasons reported for not having an improved latrine were the lack of available water for sanitation and financial difficulties for funding it. Government financial aids for latrine construction were reported to be insufficient to cover the construction costs for an improved latrine. As explained by a frontline health worker, it was common for people to start latrine constructions by building the latrine shed and slab, so that it would look like latrines were completed and government aids could be cashed, despite the latrine pits not having been dug due to a lack of sufficient funds.

“*[Families] don’t have enough money to make separate facilities for the animals*” *(Frontline health worker, Ghatol)*

*“There is no water here for drinking purpose, so how will the people here use it for toilets?” (Frontline health worker, Kushalgarh)*

*“Most of the toilets are halfway under construction and incomplete. The old people do not like to go, and the young people want but say that the toilets are not in working condition. There is a nearby drain where they defecate. And money is also too little, 12000 rupees [£130] in total [provided by the government fund], so people have started to construct it a little bit and left it as it is. Here, the toilets are only for namesake [for the official records]. No toilet is in proper condition.” (Frontline health worker, Ghatol)*

### Household-level socio-cultural factors: unsafe hygiene behaviours that may pose additional infection risks

Several unsafe hygiene behaviours, defined as domestic and personal hygiene behaviours that may pose additional risks of exposure to faecal pathogens and enteric infections in infants, were captured.

#### Unsafe food hygiene behaviours

There was a local preference for cooking and eating practices that may present an increased risk of faecal exposure to infants. While the use of off-ground gas-stoves would potentially decrease the risk of food contamination by separating the process of food preparation from the dirt floors and not having to use animal excreta (cow dung) as fuel, it was seldom used. A preference for clay-stoves strongly remained.

“*Chulha [clay-stove] is cheaper than LPG [gas-stove] and makes tastier rotis [flat bread]” (Mother, Kushalgarh)*

The cultural habit of eating by hand and sitting on the floor during meals could increase the risk of infection through repeated contact with the dirt floors and mouthing of soiled hands and food while eating. During infant feeding episodes, weaned infants were observed to eat mainly self-feeding while sitting on the dirt floors, with constants floor-to-hands, and hands-to-mouth contacts. Children’s hands were not seen to be washed before any of the 24 feeding events that were captured. Since children ate more frequent and smaller meals in comparison to adults, they often ate stale food which had been cooked in the morning and stored throughout the day until the evening. Cooked food was mostly stored covered with a plate to avoid flies, but not refrigerated or re-heated before feeding infants, despite being recommended for food safety.

#### Unsafe personal hygiene behaviours

Bathing habits potentially exposed infants to faecal pathogens through the use of surface water for bathing. Mothers reported preferring surface water bodies such as streams and ponds (when these were available) for bathing and doing laundry, due to the abundance of water that facilitated the tasks, rather than using groundwater from hand-pumps. Surface water bodies are usually more highly contaminated than groundwater sources due to the widespread open defecation practices [47], but this was not perceived as a risk. Bathing of infants was reported to be carried out daily. During the first months, new-borns were washed with a clean wet cloth, but once children were mobile, they were observed to bathe and play in the local surface waters. Soap bars were observed and reported to be always used during bathing and laundry events. However, even though soap was commonly used for bathing and laundry and considered cheap, locals reported seldom using it in other instances such as handwashing or washing kitchen utensils. In 86% of the households, at least one opportunity for handwashing before eating, cooking, feeding infants or touching animals was observed and soap not used for handwashing. Kitchen utensils were observed to be washed mostly only with water, and sometimes with mud or ash, which would potentially add another pathway of faecal pathogen transmission.

*“We wash our hands and pots [kitchen utensils] sometimes with water only, sometimes with soil. Also sometimes with soap, but not so much because we are not habitual. We use it [soap] in occasions such as washing clothes. We use it [soap] only when are hands are very dirty like after making dung cakes” (Mother, Ghatol)*

#### Unsafe water treatment strategies

In some of the study villages (particularly those in the semi-arid Kushalgarh area), groundwater from hand-pumps was reported to dry up or appear murky during the dry season.

“*The major problem is of water. From where do we get water for everyone? We have electricity and roads in our village. Our only concern is drinking water*.” *“Water is too little compared to the demand. Everyone goes to the same hand-pump to fetch water” (Mothers, Kushalgarh)*

When facing water scarcity, there was a lack of knowledge and clarity on what water sources or treatment techniques were safer. When hand-pump water was scarce, people reported either walking to find a not-depleted hand-pump, or resorting to drinking from unimproved open wells, which they preferred to avoid drinking murky water from the hand-pumps, which was perceived to be dirty. Open wells however, were observed to be contaminated from spilt water, algae, animal excreta and other objects thrown into the well.

*“If hand-pump water is not clean, we consume from wells. The well water is the best in summers, but here there is less number of wells so we have to drink unclean water*” *(Mothers, Kushalgarh)*

The belief that filtering water through a cloth would make it safe to drink was widespread. Over half the families (57%) were observed to use a cloth to sieve the drinking water when it appeared mixed with mud, as a water treatment strategy. The cloths used for filtering water were observed to be old clothing pieces, sometimes visibly dirty (i.e. stained t-shirts). The designated cloths were repeatedly re-used as filters and air dried in between. Boiling of water as a treatment was reported to be rare.

“*We tell people to sieve water with the cloth to get rid of soil and animals in it*. *Not filtering hand-pump water is the major cause of infection*”. *(Frontline health workers, Kushalgarh)*

#### Infants’ close contact with livestock

Animals were observed to be core subjects in the daily lives of rural villagers. Not only in a practical sense as part of their livelihood, but also at an emotional level, with feelings of affection, and sacredness towards them. As such, they were not perceived as potential vectors of infection transmission.

Livestock’s by-products such as milk (used to feed infants) and cow dung (used as a fertilizer, as construction material, and as fuel for cooking) were essential parts of the local livelihood. Cows were also involved in religious celebrations. Livestock ownership was also reported to be associated with higher social status, since they constituted significant initial investments for the family economy. Animals were often kept inside the house (sometimes in the same room) during the night to avoid getting them stolen or cold. All family members were seen caring for the animals, and children often playing with them, particularly with small animals. Animal contact was not observed to trigger handwashing during any of the observed events. Animals were reported to be “friends” for children, and not perceived as a potential faecal exposure pathways or infection risk. Health workers recognised that animals could act as vectors of infection transmission, but counter-argued that livestock played an essential role for the community.

“C*hildren play [with animals], and they become friends. He can’t get ill by playing with a baby goat”. (Mother, Kushalgarh)*

“*We cannot tell people to not keep animals as it is their livelihood. The cow is used in farming and is also used for manure. Also, the cows and buffaloes give milk which is also used*.” *(Frontline health workers, Ghatol)*

### Household-level demographic factors: division of childcare tasks and the role of different carers on child hygiene

Most families lived in a joint family model, where multiple generations lived together. Extended family members and dwelling neighbours were often seen helping each other with childcare and domestic responsibilities. During the first few months after birth, mothers took a prominent role for childcare and breastfeeding. However, the need to quickly attend to agriculture tasks, which were mainly carried out by young adults (male and females during parenting age) meant that childcare was often taken over by others that were not engaged in agriculture work as soon as infants started to gain mobility and wean to solid food. Grandparents and other senior neighbours were often seen being in charge of infants’ feeding and bathing while parents were engaged in agriculture. Older siblings and other children from neighbouring houses were seen playing with infants around the dwelling compound. Therefore, grandparents and older children had a prominent role in childcare, feeding and hygiene for several hours a day. Infants were often seen to be fly-ridden or with visibly dirty hands, clothes or face (70% of infants observed). However, mothers reported difficulties keeping track of the infant’s exposures to dirt, since they spent long hours working in the fields and childcare often relied on others for childcare.

*“At times children eat mud and clay. They roam around the whole day under the sun and they eat and drink anything which leads to diarrhoea. We go to work every day so we are unaware of their act and only come to know about it when we come back from work” (Mothers, Kushalgarh)*

An exception to this childcare pattern was observed in *Pucca* households, where mothers were from higher social castes and whose husbands were able to provide an additional income, which meant that mothers did not need to attend to agriculture and were able to be housewives and devote their time fully to house chores and childcare, as they reported.

### Community-level environmental factors: villages’ natural and built environment

The geographical location and climate marked by strong seasonal variations in water availability, particularly in Kushalgarh villages that were not canal-irrigated, contributed to a perpetual and worsening issue of water scarcity, considerably hindering progress in local WASH conditions. Although seasonality caused large variations in the availability of surface water in Ghatol villages, groundwater resources remained available throughout the year. In Kushalgarh, however, the impact of seasonality on their groundwater resources during the dry season depleted several of their water resources. Public water supply infrastructure was insufficient to cover drinking and sanitation needs throughout the year, as the groundwater resources have been classified as “semi-critical” by the State Ground Water Department [48].

### Community-level economic factors: local livelihood and employment

The local opportunities for a financially stable livelihood were very limited, particularly in study villages of the semi-arid area (Kushalgarh), which had very few local industries and a water-scarce agricultural land. Sustenance agriculture was the main form of livelihood but given its climate dependence, other forms of stable income were commonly sought among local men (Table 3). In Ghatol villages, because of its proximity to the district capital and to major district roads, men often had an additional income from working at a factory or shop. Among higher-caste families, 100% of adult males were employed with local jobs or jobs abroad. In Kushalgarh villages however, where the totality of the population belonged to tribal castes, families solely relied on sustenance agriculture. Given the lack of an irrigation canal in Kushalgarh and the lack of job opportunities, during the months of drought when crops could not be harvested, parents often had to migrate for 3–4 months to nearby states for temporary jobs. Temporary seasonal migration placed an additional burden on parents to maintain a stable and clean environment for infant caring practices.

**Table 3:** Main livelihoods of the household observation participants

Households visited	Ghatol	Kushalgarh
	Overall (*N* = 21)	Overall (*N* = 21)
ST/SC caste, n (%)	17 (81%)	21 (100%)
Father’s occupation, n (%)		
Agriculture only	6 (29%)	1 (5%)
Agriculture + short-term seasonal migration	0 (0%)	18 (86%)
Long-term migration: job abroad	3 (14%)	0 (0%)
Agriculture + local job	12 (57%)	2 (9%)
Mother’s occupation, n (%)		
Housewife	6 (29%)	3 (14%)
Housewife + helps in agriculture	15 (71%)	18 (86%)

Overall, the general state of poverty (particularly for tribal castes) hindered the capacity to improve hygienic environments and may contribute to pushing domestic and child hygiene concerns down the list of priorities, below securing a stable livelihood. In this poor villages, developments in the material circumstances were seen to be highly dependent on government schemes, and insufficient public financial resources to improve water, sanitation and housing impeded progress in domestic and child hygiene.

### Community-level socio-cultural factors: perceived lack of control to address child enteric infection risks

Communities mostly recognised that a lack of hand hygiene, “bad” eating and drinking habits, ingesting soil or animals’ excreta were enteric infection risks for young children, however they failed to interrupt the specific exposure pathways to faecal pathogens (i.e. exposure to dirt and animal faeces), and sanitize the environment (i.e. handwashing with soap, boiling water).

*“[Infants] get infection in the stomach as they don’t wash their hands and roam around the whole day. They get infected because of their bad eating and drinking habits. Small children are unaware of hygiene habits ( …) and might be eating the mud (..), eating stale food”. (Mothers, Ghatol)*

By contrast, only a few caregivers reported not knowing what the causes of diarrhoea might be, and simply referring to the doctors for treatment when symptoms presented.

*“We have no idea [what things can cause diarrhoea]. On consulting the doctor he provides treatment to our kids. He gives us medicines and we pay them. He assures us that our child will heal. He doesn’t brief us with the cause of the illness. If the treatment does not work, we take our child back to the doctor” (Mothers, Kushalgarh)*

Despite their knowledge on infection risks, caregivers reported a perceived lack of control to interrupt faecal exposures and improve child hygiene in the farming environments where they lived. When discussing enteric infection risks, mothers frequently reported that “*it happens, but what to do?”*, a quote that exemplified the perceived lack of self-efficacy in avoiding child faecal exposures due to the wider factors that shaped their environment.

“*At times children eat mud and clay. Our kids can even put their hands in the cow dung, and it has so many germs which is the cause of their illness. If we do not have farms and animals around us, then ours kids will be also clean. Even if we try to keep our kids neat and tidy, they again get themselves in dirt and mud”. “It happens, but what to do?” (Mothers, Kushalgarh)*

### Community-level institutional factors: unreliable local governments fail to deliver public services and infrastructure

Mistrust in political leadership was often captured throughout the study communities. Villagers repeatedly brought up the topic of misuse of public funds and resources due to political corruption. In some villages, locals reported that Sarpanch elections were often bribed, that developmental aid funds were favourably distributed to friends and relatives only, or that village funds were embezzled. Weak monitoring of frontline health workers roles sometimes also hindered the delivery of child health and hygiene rural programmes. Weak governance monitoring in rural remote areas remained a remarkably relevant factor hindering progress in the village and household-level WASH infrastructure and childcare services, often funded through public aids. Some caregivers reported not trusting the public health services provided for child health and did not trust the advice from frontline health workers.

“*I sometimes give advice, support and suggestion but some people from the community tell me bad words.” (Frontline health worker, Kushalgarh)*

*“No good services provided there, [the rural childcare centre] is only there for the government records, they don’t provide good support”. (Mothers, Ghatol)*

Corruption and weak governance may have had a significant emotional impact at a population level. Exposure to unfairness and dishonesty in the leadership roles leads to passive and resigned communities. The community’s lack of initiative and perceived self-efficacy to improve the water, sanitation and hygiene conditions was also hindered by a sense of state paternalism: the belief that it was the states’ responsibility to protect people, and that solutions were not within the reach of individuals.

### Holistic conceptual mapping of infant enteric infection drivers

**Fig. 2:**
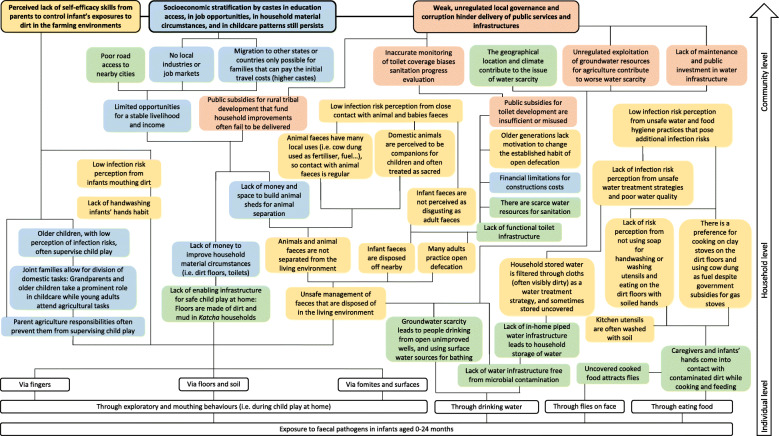
Conceptual map of infant enteric infection drivers in rural Banswara

The list of themes presented insofar provided a fragmented explanation of the complexity of the enteric infection drivers. Figure 2 presents the conceptual map that considers the interplay between the environmental, socio-cultural, economic-demographic, and institutional factors identified (each represented by a coloured concept box). By organising the interlinked factors across different levels in a conceptual map, some overall patterns were identified. For instance, political corruption in the delivery of public services, harsh living conditions and low socioeconomic status, and the inequalities by castes seemed to be key factors shaping the caregivers’ motivation, initiative and perceived self-efficacy to improve the hygienic conditions of their living environment, ultimately determining several key enteric infection risk factors for infants.

## Discussion

As the burden of child enteric infections and malnutrition remain a pressing concern for India’s development goals, and current WASH RCTs have mostly failed to identify links with improved child health outcomes, this study used mixed-methods to develop an in-depth holistic understanding of the multiple and multifaceted drivers to child enteric infections, using rural tribal villages of Banswara as a case study.

### New insights into the enteric infection drivers in rural India

In the study villages of rural Banswara, the typical *Katcha* household material circumstances (i.e. dirt floors, no in-home water access, no toilets, no animal sheds), and several unsafe domestic hygiene behaviours that were captured (i.e. unsafe water treatment, unsafe food hygiene habits, lack of soap use for handwashing and cleaning utensils, close contact between infants and animals and dirt …) introduced additional faecal exposure risks to infants. Several of these exposures, such as soil ingestion during child play, are infant-specific and only recently have studies started to recognise them as potential infection transmission pathway for infants [49, 50]. In fact, mouthing of soiled hands and direct ingestion of soil has already been proven to be the most dominant infection transmission pathways for infants under 2 in rural Bangladesh, above drinking and eating contaminated water and food [51]. However, the infants’ exploratory and mouthing behaviours are key for child development and hardly modifiable, so it is the household dirt environments that need to be addressed. For example, household ownership of domestic animals, particularly poultry, contributes to the contamination of child play spaces with animal faeces and it has been linked to increased odds of child diarrhoea [52]. Recent and ongoing trials are testing the effects of providing play-pens, play-mats and improved household flooring to reduce infants’ exposures to dirt and animal faeces during play [26, 53–56]. Preliminary results suggest potential benefits to child health and clear additional benefits for caregivers. Mothers have reported that playpens make caregiving easier, but have also reported difficulties in maintaining the child play spaces clean due to the farming surrounding environment [55, 56], similar to what we captured in Banswara. In addition, improving a single aspect of the household infrastructure, the flooring, was seen to have a positive effect on several domestic hygiene behaviours overall, as it may revalorise how people perceived the domestic environment [53, 57].

In addition to important individual and household-level enteric infection drivers, several wider structural factors were also identified. For instance, while parents were observed to carry out several unsafe domestic hygiene behaviours that may introduce additional faecal exposures, interviews and discussions revealed that parents often reported a perceived lack of self-efficacy to block infant’s exposures to dirt due to the rural and farming environment in which they lived (i.e. *“What to do?*”). The parents’ perceived lack of control and resignation to improve their surroundings and interrupt infants’ exposures to faecal pathogens may have been largely influenced by the hampering of local geographical (water scarce area), socio-economic (general state of poverty and caste inequalities) and institutional (political corruption and state paternalism) contextual factors. Further studies on the tribal communities’ social norms and values may provide valuable new insights into latent factors contributing to disease burden. The role of such wider-level factors may need to be better recognised when addressing complex problems. For example, studies have found that a high socio-economic status threshold will need to be surpassed before child undernutrition and stunting can be eliminated, independent of any WASH-specific improvements [58], and yet tribal castes still faced evident socio-economic limitations. Corruption has been suggested to be the biggest barrier to improving health in developing countries [59], and yet, rates of absenteeism among community health workers in rural India may be as high as 60% [60]. Transparent governance remains a fundamental challenge at the global scale, and WASH programming would benefit from acknowledging these limitations and designing programmes that reduce incentives and opportunities for corruption from the outset [60]. In fact, international organisations are now beginning to design WASH interventions that are explicitly and uniquely aimed at addressing the governance systems’ weakness, focusing on strengthening the network of actors and factors that deliver services rather than on the provision of infrastructure or behaviours [61]. The study villages in Banswara would benefit from systems-strengthening protocols that establish monitoring and accounting strategies of the Gram Panchayats, as misuse of funds by Panchayati institutions has been found to continue to hinder rural development [62].

### Holistic understanding of the system

Interlinking the individual, household and community-level factors in the conceptual map helped us understand how wider-level factors such as the socio-economic limitations, caste inequalities, and corruption may have trickle down effects in the community’s motivation and perceived self-efficacy for improving hygiene levels around children. Overall, the conceptual map stressed the impact of wider-level cultural, economic and institutional factors in shaping barriers to hygiene development and ultimately enteric infection risks. Previous use of holistic conceptual maps also highlighted the impact of institutional and cultural patterns: In Mexico, sanitation development was seen to be influenced by socio-cultural dynamics of bribery and machismo [44]. Under the current POSHAN Mission to reduce child stunting in India, inter-ministerial convergence efforts to address the complex drivers of child infections and stunting are already in place [63]. Nevertheless, there is a need to accelerate improvement trends. Our findings suggest that to do so, convergence efforts will need to extend beyond programmes addressing the most immediate drivers such as water, sanitation, hygiene and nutrition, and will need to incorporate programmes targeting the wider-level social determinants of health. Particularly, addressing the social inclusion, weak governance and livelihood insecurity pressures faced by the tribal communities in Banswara may prove crucial to accelerate reduction of infection rates and stunting.

A progression toward more comprehensive integrated approaches is being seen in the WASH sector, and the use of holistic explorations of factors across domains and socio-ecological levels, or the use of conceptual maps to visualise the network of interlinked factors may prove useful strategies to adopt such integrated approaches. After recent trials failed to interrupt enough infection transmission pathways to improve child health outcomes, the marching orders set for the sector emphasised the need for a holistic understanding of all key pathways for contamination of the environment and infant exposure to faecal pathogens [28]. The role of anthropologists, psychologists and other professionals in interdisciplinary teams for the design and delivery of WASH programmes is increasingly recognised [64]. Further efforts towards integrated assessments of exposures and holistic approaches to solutions in complex settings are needed to achieve the synergistic SDGs. Efforts towards improving WASH have proven to lead to wide-ranging benefits ultimately addressing all 17 SDGs [65]. The 17th SDG outlines the need to form partnerships to achieve the goals. Similarly, the holistic conceptual map in this case study warrants for coordinated action, drawing attention to the interlinkages between wider-level socio-economic and institutional challenges addressed in SDG1, SDG10, SDG16 (No Poverty, Reducing Inequalities, and Strengthening Institutions, respectively), and family and individual-level challenges addressed in SDG2, SDG3 and SDG6 (Zero Hunger, Good Health, and Clean Water and Sanitation, respectively) [66].

### Limitations

This exploratory case study design is limited in its generalisability, as the particular risk factors, behaviours or attitudes captured may be context-specific. However, it serves as an exemplary case for numerous similar rural tribal villages in India. It responds to the aim of an in-depth holistic understanding, rather than in breadth. Authenticity of findings was sought through triangulation of data on each concept until the point of theoretical saturation. Credibility was sought through the presentation of vivid data with detailed text, observations and pictures to draw conclusions [67]. This study was limited in the duration of household observations due to feasibility reasons, but the homogeneity across study villages meant that information saturation was reached in terms of capturing the diversity of domestic and childcare practices and beliefs.

Overall, the integrated holistic representation is a novel way to assess infection risk factors that is closer to a realistic model. The study approach, which develops a comprehensive integrated and in-depth knowledge on the wider enteric infection determinants for infants, might prove useful towards *transformative* WASH intervention design. Furthermore, as posed by Chambers, the development of a deep understanding in grounding realities may be a key approach for timely and relevant learnings for policy. As randomised control trials are the gold standard for accurate, replicable, and rigorous research, they more often fail to equally address timeliness of recommendations for policies, given the large time-scales required for RCT conclusions to be reached [68].

## Conclusions

After the recently failed trials, emerging evidence is recognising the need to address the multi-scalar and multi-faceted factors that influence WASH [69]. Our findings suggest that future research towards *transformative* WASH needs to look beyond WASH factors as currently defined, beyond improvements in household material circumstances and domestic hygiene behaviours, and recognise the impact of the wider-level factors (cultural, socio-economic and institutional) contributing to enteric infections in infants. Through holistic conceptual mapping, we established the influence of the socioeconomic gap between castes, the institutional corruption in the delivery of public services and infrastructure, and the limited livelihood opportunities on the communities’ resignation and perceived lack of self-efficacy to improve hygiene levels from their infants’ surroundings. Empowering community actors and restoring motivation and self-efficacy under a common vision and commitment for progress in hygiene and child health may be key aspects of future WASH interventions. To achieve the Sustainable Development Goals by 2030, future WASH interventions are likely to require an integrated understanding of the complex and interlinked factors across socio-ecological levels and domains, including individual, technical, and behavioural aspects, as well as wider sociocultural, economic, and policy aspects.

## Data Availability

The data used and analysed in the current study are available from the corresponding author on reasonable request.

## Abbreviations

FGD: Focus Group Discussion
KII: Key Informant Interview
NGO: Non-governmental Organisation
SDG: Sustainable Development Goals
ST/SC: Scheduled Tribes and Scheduled Castes
WASH: Water, Sanitation and Hygiene.
